# Unraveling the Complex Web of Fibromyalgia: A Narrative Review

**DOI:** 10.3390/medicina60020272

**Published:** 2024-02-04

**Authors:** Sarah Al Sharie, Scott J. Varga, Lou’i Al-Husinat, Piercarlo Sarzi-Puttini, Mohammad Araydah, Batool Riyad Bal’awi, Giustino Varrassi

**Affiliations:** 1 Faculty of Medicine, Yarmouk University, Irbid 21163, Jordan; 2 Department of Neurology, OhioHealth Mansfield General Hospital, Mansfield, OH 44903, USA; scott.varga@ohiohealth.com; 3 Department of Clinical Sciences, Faculty of Medicine, Yarmouk University, Irbid 21163, Jordan; loui.husinat@yu.edu.jo; 4 Rheumatology Unit, Internal Medicine Department, ASST Fatebenefratelli-Sacco, University School of Medicine, 20157 Milan, Italy; piercarlo.sarziputtini@gmail.com; 5 Department of Internal Medicine, Istishari Hospital, Amman 11942, Jordan; mohaari98@gmail.com; 6 Department of Family Medicine, Jordan Royal Medical Services, Amman 11855, Jordan; batool.balawi@gmail.com; 7 Paolo Procacci Foundation, 00193 Rome, Italy

**Keywords:** fibromyalgia, chronic pain, diagnosis, management, emerging treatments

## Abstract

Fibromyalgia is a complex and often misunderstood chronic pain disorder. It is characterized by widespread musculoskeletal pain, fatigue, and heightened sensitivity, and has evolved in diagnostic criteria and understanding over the years. Initially met with skepticism, fibromyalgia is now recognized as a global health concern affecting millions of people, with a prevalence transcending demographic boundaries. The clinical features and diagnosis of fibromyalgia encompass a range of symptoms beyond pain, including sleep disturbances and cognitive difficulties. This study emphasizes the importance of a comprehensive evaluation for accurate diagnosis, considering the shift from tender point reliance to a more holistic approach. Etiology and pathophysiology involve genetic predisposition, neurotransmitter dysregulation, central sensitization, and immune system involvement. Risk factors such as gender, age, family history, and comorbid conditions contribute to susceptibility. The impact on quality of life is profound, affecting physical and social aspects, often accompanied by mood disorders. Management approaches include pharmacological interventions, non-pharmacological therapies, lifestyle modifications, and alternative treatments. This study also delves into emerging research, exploring advances in neurobiological understanding, brain imaging, genetic markers, glutamate modulation, cannabinoids, gut microbiome, and digital health tools for fibromyalgia management. Overall, this study provides a nuanced and up-to-date overview of the complexities surrounding fibromyalgia, aiming to enhance understanding and support for individuals grappling with this challenging condition.

## 1. Introduction

Fibromyalgia, a term coined in the early 1970s, represents a complex and challenging clinical entity that extends beyond the boundaries of traditional medical classifications [1]. In the realm of chronic pain disorders, fibromyalgia stands as a perplexing and often misunderstood condition, characterized by widespread musculoskeletal pain, tenderness, and a constellation of associated symptoms [2].

At its core, fibromyalgia is a chronic pain syndrome characterized by widespread musculoskeletal pain, fatigue, sleep disturbances, and heightened sensitivity to tactile stimuli [3]. One of the hallmark features is the presence of tender points on the body, as defined by the American College of Rheumatology (ACR) [4]. However, the understanding of fibromyalgia has transcended a mere constellation of symptoms; it encompasses a broader spectrum of physiological and psychological intricacies [5].

The definition of fibromyalgia has undergone notable revisions over the years, reflecting the evolving understanding of the condition [6]. Initially perceived primarily as a rheumatic disorder, it is now recognized as a disorder of pain processing and central nervous system sensitization [6,7]. The diagnostic criteria have shifted from reliance solely on tender points to a more comprehensive evaluation, considering the widespread nature of pain and associated symptoms [8].

The concept of fibromyalgia can be traced back to the early 19th century when physicians described a condition known as muscular rheumatism [1]. However, it was not until the late 20th century that fibromyalgia emerged as a distinct entity. In 1990, the ACR introduced the first set of classification criteria, formalizing fibromyalgia as a recognized medical condition [9].

Historically, fibromyalgia was often met with skepticism within the medical community, with some dismissing it as a psychosomatic disorder [10]. This skepticism, rooted in a lack of objective diagnostic markers, hindered the acknowledgment and understanding of fibromyalgia [10]. Over time, however, advancements in research and a growing body of evidence have elucidated the complex interplay of biological, psychological, and social factors contributing to the syndrome [11,12].

Once considered a rare and enigmatic condition, fibromyalgia has gained recognition as a prevalent health concern on a global scale [13]. Epidemiological studies reveal a staggering prevalence, with estimates suggesting that millions of individuals worldwide are affected by fibromyalgia [14]. The prevalence is not confined to a specific demographic, transcending age, gender, and socio-economic status [14].

The epidemiology of fibromyalgia paints a nuanced picture, showcasing its impact on diverse populations [15]. Women are disproportionately affected, with a prevalence several times higher than that in men [16]. The condition often manifests during middle adulthood, although it can affect individuals of any age, including adolescents and the elderly [17].

This comprehensive review delves into the multifaceted landscape of fibromyalgia, aiming to provide a nuanced understanding of its intricacies. From its historical roots to contemporary research, this exploration seeks to shed light on the evolving understanding of fibromyalgia.

## 2. Clinical Features and Diagnosis

Fibromyalgia presents a clinical panorama marked by a myriad of symptoms, often making the diagnosis a complex process that requires a comprehensive evaluation of the patient’s medical history, physical examination, and consideration of associated factors [18,19,20].

The hallmark symptom of fibromyalgia is widespread, chronic musculoskeletal pain. Pain is typically present on both sides of the body, above and below the waist, and along the spine [21,22]. The pain is often described as a deep, persistent ache and may vary in intensity [22]. Patients commonly experience profound fatigue, regardless of the quantity or quality of sleep [23].

Sleep disturbances are pervasive in patients affected by fibromyalgia. They frequently report difficulties falling asleep, staying asleep, or experiencing restorative sleep [24]. These disturbances contribute to the cycle of pain and fatigue [24].

Many individuals with fibromyalgia report cognitive difficulties, often referred to as “fibro fog”. This includes problems with concentration, memory, and the ability to perform mental tasks [25]. Moreover, fatigue accounts for one of the most common symptoms of fibromyalgia [26].

The presence of tender points is a characteristic feature, although it is no longer the sole criterion for diagnosis [27]. These tender points are specific anatomical sites where pressure elicits pain [27]. Historically, the ACR defined 18 tender points symmetrically distributed across the body; however, the diagnostic approach has evolved to encompass a more holistic evaluation [28].

Patients may also experience a range of other symptoms, including headaches, irritable bowel syndrome (IBS), temporomandibular joint (TMJ) disorders, anxiety, and depression [29,30,31].

**Figure 1:**
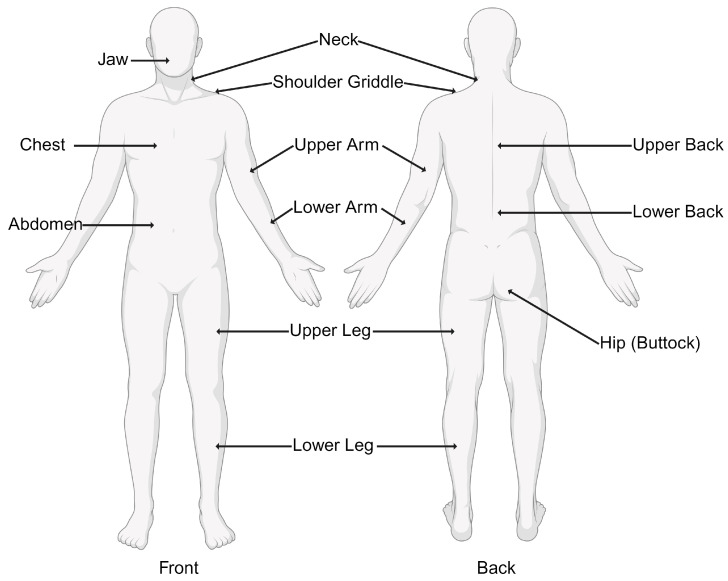
Nineteen tender points used by the Widespread Pain Index (WPI) in the diagnosis of fibromyalgia.

The diagnosis of fibromyalgia has evolved from the initial emphasis on tender points to a more comprehensive and inclusive approach [6]. The ACR has updated its diagnostic criteria to better capture the diverse manifestations of fibromyalgia [32]. The current criteria, established in 2010, include:

1. Widespread Pain Index (WPI): This involves assessing pain in 19 specified body areas over the past week. The areas include the neck, shoulders, chest, arms, lower back, hips, and legs. Figure 1 demonstrates the 19 specific tender points used in the diagnosis of fibromyalgia.

2. Symptom severity (SS) score: In addition to the WPI, the SS score considers the severity of other symptoms such as fatigue, sleep disturbances, and cognitive difficulties. Table 1 demonstrates SS score calculation variables.

To meet the diagnostic criteria, a patient must have widespread pain (WPI ≥ 7) and SS score ≥ 5 or WPI of 3–6 and SS score ≥ 9 (29).

## 3. Differential Diagnosis

Given the overlapping nature of symptoms, fibromyalgia can be challenging to distinguish from other conditions [33]. A thorough differential diagnosis is essential to rule out similar disorders, including rheumatoid arthritis (RA), systemic lupus erythematosus (SLE), and inflammatory arthritis, which present with joint pain and stiffness that can mimic fibromyalgia symptoms, but their inflammatory nature is what sets it apart [34]. Moreover, chronic fatigue syndrome (CFS) can be distinguished by the fact that, unlike fibromyalgia, CFS is primarily characterized by profound fatigue and post-exertional malaise [35]. Furthermore, hypothyroidism can cause fatigue and musculoskeletal pain, resembling fibromyalgia symptoms which can be excluded by a thorough thyroid investigation [36].

Accurate diagnosis involves a thorough evaluation by a healthcare professional, often a rheumatologist, who considers the patient’s symptoms and medical history and excludes other potential causes of pain and fatigue [37]. A multidimensional approach to diagnosis ensures a more accurate and nuanced understanding of fibromyalgia in the context of an individual’s overall health [38].

## 4. Etiology and Pathophysiology

Fibromyalgia’s etiology and pathophysiology are intricate and multifaceted, involving a complex interplay of genetic, neurological, and immunological factors [18]. While the precise mechanisms remain incompletely understood, contemporary research has provided valuable insights into the contributors to the development and perpetuation of fibromyalgia [39].

### 4.1. Genetic Factors

Genetic predisposition plays a significant role in a person’s susceptibility to fibromyalgia [40]. Studies have identified specific genetic markers associated with an increased risk of developing the condition [41]. The heritability of fibromyalgia is estimated to be around 50%, indicating a substantial genetic influence [42]. Variations in genes involved in pain perception, neurotransmitter regulation, and immune function have been implicated [43].

The identification of genetic factors provides a foundation for understanding the hereditary nature of fibromyalgia, but it is important to recognize the interaction between genetics and environmental factors [40]. Environmental triggers, such as physical trauma, infections, or stressful life events, may act as catalysts in individuals with a genetic predisposition, contributing to the onset of fibromyalgia [44].

A study by D’Agnelli et al. [45] suggests that potential candidate genes associated with fibromyalgia include SLC64A4, TRPV2, MYT1L, and NRXN3 and that a gene–environment interaction, involving epigenetic alterations, has been proposed as a triggering mechanism. Moreover, they have demonstrated that fibromyalgia exhibits a hypomethylated DNA pattern in genes related to stress response, DNA repair, autonomic system response, and subcortical neuronal abnormalities.

### 4.2. Neurotransmitter Dysregulation

Neurotransmitter dysregulation is a central feature in the pathophysiology of fibromyalgia, impacting the processing of pain signals in the central nervous system [46]. Several neurotransmitters, including serotonin, norepinephrine, and dopamine, are implicated in the altered pain perception observed in fibromyalgia patients [46].

Low levels of serotonin have been consistently observed in fibromyalgia [46]. A case–control study focusing on fibromyalgia involved 35 healthy women (Group I) as controls and 130 women with fibromyalgia (Group II) [47]. The study found a significantly lower serum serotonin level in fibromyalgia patients compared to healthy individuals and a positive significant correlation was observed between serotonin levels and tender points in fibromyalgia patients, suggesting associations between fibromyalgia and certain demographic factors, hematological platelet indices, and serotonin levels.

Moreover, the dysregulation of norepinephrine, which plays a role in the body’s stress response and pain modulation, is also evident in fibromyalgia [48]. This dysregulation may contribute to the heightened sensitivity to pain and the characteristic fatigue experienced by fibromyalgia patients [48].

A prospective double-blind controlled study involving 20 fibromyalgia patients, 20 rheumatoid arthritis patients, and 20 healthy controls aimed to assess norepinephrine-evoked pain by injecting norepinephrine and a placebo (saline solution) into separate forearms [49]. The study showed that 80% of fibromyalgia patients experienced norepinephrine-evoked pain, compared to 30% of rheumatoid arthritis patients and 30% of healthy controls. The intensity of norepinephrine-evoked pain was significantly greater in fibromyalgia patients (2.5 ± 2.5) compared to rheumatoid arthritis patients (0.3 ± 0.7) and healthy controls (0.3 ± 0.8) with a p-value less than 0.0001 suggesting that fibromyalgia patients exhibit heightened sensitivity to norepinephrine-induced pain compared to the other groups studied [49].

Also, dopamine has been implicated in the emotional aspects of fibromyalgia as the dysregulation of dopamine pathways may contribute to the mood disorders often observed in fibromyalgia patients [50]. The findings of a study suggest that fibromyalgia patients experience disrupted release of endogenous dopamine in response to both experimental pain and nonpainful stimulation in the basal ganglia [51]. This dysfunction in dopaminergic neurotransmission may explain the main clinical symptoms of fibromyalgia, e.g., widespread pain and bodily tenderness. It also raises the possibility that other symptoms of fibromyalgia may also result from this abnormality [51].

### 4.3. Central Sensitization

Central sensitization is a key concept in understanding the amplification of pain signals in fibromyalgia [52]. It involves an abnormal response of the central nervous system to stimuli, leading to an exaggerated and prolonged pain experience [53].

It is linked to alterations in the function of N-methyl-D-aspartate (NMDA) receptors and an imbalance in excitatory and inhibitory neurotransmitter systems [54]. This phenomenon contributes to the widespread and persistent pain experienced by individuals with fibromyalgia [53].

### 4.4. Immune System Involvement

Emerging evidence suggests that immune system dysregulation and abnormalities in immune function, including increased levels of inflammatory cytokines, may contribute to the pathophysiology of fibromyalgia [55]. A study discussed the reduced immune system responsiveness in fibromyalgia and compared the two groups [55]. The characteristics of the fibromyalgia group included higher pain levels, greater fatigue, lower quality of life, and a higher prevalence of depression. It also exhibited altered responses to nociceptive tests. Moreover, the study analyzed monocyte characteristics and peripheral blood mononuclear cell (PBMC) responses after stimulation. The fibromyalgia group showed differences in the percentage of cells with monocytic properties, particularly under unstimulated conditions. Additionally, there were variations in CD14 and CD16 cell percentages and mean fluorescence intensity (MFI) after stimulation. PBMC cultures from both groups exhibited a similar capacity to secrete IL-6 and IL-10 after stimulation, with a tendency for a lower stimulation index for IL-6 in the fibromyalgia group. B-cell and T-cell characteristics were also examined, revealing lower percentages of CD19+ B-cells in the fibromyalgia group. Both groups responded similarly to stimulation, with an increase in CD69+ cells. The study also investigated cytokine secretion related to T-helper subsets and T-cytotoxic cells, finding lower stimulation indices for IFN-γ in the fibromyalgia group. Correlation analysis revealed a negative correlation between the IFN-γ stimulation index and the cold pain threshold in the fibromyalgia group.

### 4.5. Oxidative Stress

Oxidative stress has been explored as a potential contributor to the pathophysiology of fibromyalgia [56]. Mitochondrial dysfunction, evident in increased ROS production, has been associated with fibromyalgia, suggesting a role for disrupted energy metabolism [57]. Additionally, oxidative stress may contribute to the heightened pain sensitivity characteristic of fibromyalgia by activating nociceptive neurons and impacting pain pathways [56]. The antioxidant defenses in fibromyalgia patients may be compromised, as evidenced by lower levels of antioxidants, further exacerbating oxidative stress [58]. The influence of oxidative stress on neurotransmitter systems implicated in pain perception and mood regulation adds another layer to the complex nature of fibromyalgia [58].

A study by Coppens et al. investigated the response of fibromyalgia patients to stress. It focused on cortisol levels and subjective stress in response to the Trier Social Stress Test (TSST), considering the influence of early childhood adversities (ECA). Key findings included fibromyalgia patients showing blunted cortisol responsivity to stress compared to controls, especially when ECA was accounted for [59].

## 5. Risk Factors

Understanding the risk factors associated with the development of fibromyalgia provides valuable insights into its etiology and helps identify individuals who may be more susceptible to this condition.

### 5.1. Gender and Age

Fibromyalgia exhibits a striking gender disparity, with a significantly higher prevalence in women compared to men [60]. Studies consistently report that approximately 80–90% of individuals diagnosed with fibromyalgia are women [60]. The reasons behind this gender predominance are not fully understood but may involve hormonal, genetic, and psychosocial factors [61]. Hormonal fluctuations, particularly during reproductive stages such as menopause, are thought to influence symptom severity and prevalence in women [60].

A study aimed to investigate the relationship between sex hormones and pain severity in individuals with fibromyalgia [62]. The findings revealed an inverse relationship between pain severity and both progesterone and testosterone levels, with progesterone showing the strongest association. Post hoc analyses indicated an interaction between cortisol and progesterone, with pain being greatest on days when progesterone was low, and cortisol was high. Overall, this suggests a complex interplay between sex hormones, cortisol, and pain severity in individuals with fibromyalgia.

While fibromyalgia can affect individuals of all ages, it most commonly emerges during middle adulthood [63,64]. The risk of developing fibromyalgia tends to increase with age, peaking in individuals between 20 and 55 years old [65]. However, cases have been reported in children and adolescents, emphasizing the importance of recognizing and understanding fibromyalgia across the lifespan [66].

### 5.2. Family History

A familial clustering of fibromyalgia cases suggests a genetic predisposition to the condition [40]. Individuals with a family history of fibromyalgia are at an increased risk of developing the syndrome themselves [2]. Genetic studies have identified specific polymorphisms and variations in genes associated with pain processing and neurotransmitter regulation that may contribute to the hereditary nature of fibromyalgia [67].

A study focused on multi-case families with fibromyalgia recruited probands meeting the 1990 ACR criteria for primary fibromyalgia and their first-degree relatives [68]. A total of 116 families were evaluated, with 342 siblings included in the assessment of sibling recurrence risk ratio (λs). The sibling recurrence risk for fibromyalgia was estimated at 27.2%, and the λs was 13.6, suggesting a strong familial aggregation of fibromyalgia. The study also conducted a genome scan, identifying several loci with nominal significance, and one region on chromosome 17p11.2-q11.2 showed suggestive linkage to fibromyalgia. The logarithm-of-odds (LOD) score for the best signal on chromosome 17p11.2-q11.2 at marker D17S2196 was 2.52, surpassing the threshold for genome-wide suggestive linkage. These findings suggest a potential genetic basis for fibromyalgia, with chromosome 17p11.2-q11.2 as a region of interest for further investigation.

### 5.3. Comorbid Conditions

Fibromyalgia often coexists with other medical and psychiatric conditions, reflecting the complex and interconnected nature of the syndrome [5]. Several comorbidities are commonly associated with fibromyalgia, including rheumatic diseases, psychiatric disorders, chronic fatigue syndrome, and sleep disorders [69]. Addressing both the primary symptoms of fibromyalgia and associated comorbidities is essential for improving the overall well-being and quality of life for affected individuals.

Furthermore, obesity is often associated with an increased risk and severity of fibromyalgia, suggesting a link between body weight and the development or exacerbation of fibromyalgia symptoms [70,71].

## 6. Impact on Quality of Life

Everyday activities such as walking, standing, and lifting may become arduous tasks, limiting the individual’s ability to engage in work, household chores, or recreational activities [72]. Poor-quality sleep impairs the body’s ability to recover, exacerbating fatigue and increasing the perception of pain [73]. The resulting sleep deficits further hinder daily functioning and contribute to a cycle of increased pain and decreased activity [73]. Moreover, stiffness and muscle weakness lead to functional limitations in joint mobility and overall physical performance [74]. These limitations may affect the ability to maintain an active lifestyle and participate in activities that were once enjoyed [74]. All of this may lead to patients limiting social interactions due to the unpredictability of symptoms, fear of judgment, or the physical toll of participating in social activities [75].

The chronic nature of the condition, coupled with the challenges in managing symptoms, can contribute to feelings of hopelessness, frustration, and helplessness and is almost always associated with depression, anxiety, and mood disorders [5]. Its associated symptoms can erode an individual’s sense of self-efficacy and self-esteem and may lead to a sense of loss and a negative impact on one’s self-image [76].

The “fibro fog” impacts various aspects of daily life [77]. This can lead to challenges in the workplace, including difficulties in maintaining regular employment, reduced productivity, and increased absenteeism [78]. Also, family members and friends may struggle to comprehend the invisible nature of the condition, leading to strained relationships [79].

## 7. Management Approaches

### 7.1. Pharmacological Interventions

Over-the-counter analgesics such as acetaminophen may provide some relief. Also, nonsteroidal anti-inflammatory drugs (NSAIDs) can help with inflammation and pain but may have limited effectiveness in fibromyalgia [80].

Tricyclic Antidepressants (TCAs) such as amitriptyline and nortriptyline are commonly used to improve sleep and reduce pain [81]. Moreover, selective serotonin reuptake inhibitors (SSRIs) and serotonin–norepinephrine reuptake inhibitors (SNRIs) including duloxetine and milnacipran may help alleviate pain and improve mood [81].

Anti-seizure medications such as gabapentin and pregabalin may help reduce neuropathic pain and improve sleep [82]. Also, muscle relaxants like cyclobenzaprine may be prescribed to alleviate muscle spasms and improve sleep quality as well [83].

While numerous studies have assessed the effectiveness of single-drug treatments, there is notably limited evidence examining the use of combination-drug therapy in individuals who do not respond to single-drug treatments. Nonetheless, small-scale studies have indicated promising results in this context [84]. It is crucial for healthcare providers to work closely with individuals to find the most effective medication with the fewest side effects for their specific symptoms and needs.

### 7.2. Non-Pharmacological Therapies

Tailored exercise programs, stretching, and strength training can help improve flexibility, reduce pain, and enhance overall physical function [85]. A Cochrane systematic review identified randomized controlled trials (RCTs) related to exercise interventions for adults with fibromyalgia. The review included 29 RCTs that assessed mixed exercise interventions, including aerobic, resistance, and flexibility exercises, against control groups or other exercise interventions. Results indicated moderate-quality evidence supporting the positive effects of mixed exercise on health-related quality of life (HRQL), pain, fatigue, stiffness, and physical function compared to control groups [86]. Also, occupational therapists can assist in finding adaptive strategies to manage daily tasks and reduce the impact of fibromyalgia on daily life [87]. Moreover, some studies support the efficacy of Hyperbaric Oxygen Therapy (HBOT) in Tender Points Count (TPC) and the enhancement of functions in fibromyalgia [88].

Cognitive behavioral therapy (CBT) aims to address negative thought patterns and improve coping mechanisms [89]. It has been shown to be effective in managing fibromyalgia-related symptoms and improving overall quality of life [89].

Some individuals find relief through acupuncture, a traditional Chinese medicine technique involving the insertion of thin needles into specific points on the body [90,91]. Moreover, massage and practices such as yoga, tai chi, and mindfulness meditation may help alleviate muscle tension, improve circulation, and reduce stress, providing relief from some fibromyalgia symptoms [92]. Furthermore, biofeedback involves learning to control physiological functions, such as heart rate and muscle tension, to improve symptoms like pain and stress [93].

### 7.3. Lifestyle Modifications

Engaging in regular, low-impact exercise, such as walking, swimming, or cycling, is essential for managing symptoms [94]. Also, establishing a consistent sleep routine, creating a comfortable sleep environment, and practicing good sleep hygiene can contribute to better sleep quality [94].

Managing stress is crucial for individuals with fibromyalgia [95]. Techniques such as deep breathing exercises, progressive muscle relaxation, and mindfulness can be beneficial [95].

A study by Vambheim et al. [96] investigating the effect of relaxation techniques on chronic pain indicates that incorporating relaxation techniques into chronic pain management programs can be advantageous. However, the efficacy of relaxation techniques tends to decrease over time, underscoring the significance of continuous practice for maintaining prolonged pain reduction.

Moreover, a well-balanced diet can contribute to overall health and may play a role in managing symptoms [97]. Some individuals find that dietary changes, such as reducing caffeine or avoiding certain trigger foods, can be helpful.

The findings of a study indicate that individuals adhering to a vegan diet experienced notable enhancements in reported pain [98]. Additionally, those following low fermentable oligo di-monosaccharides and polyols (FODMAP) diets also reported significant improvements in pain. Furthermore, they indicate that supplementation with Chlorella green algae, coenzyme Q10, acetyl-l-carnitine, or a combination of vitamin C and E demonstrated significant improvements in measures of pain.

### 7.4. Alternative and Complementary Therapies

Some individuals explore herbal supplements, such as turmeric or ginger, for their potential anti-inflammatory properties [99]. A cross-sectional survey aimed to investigate self-reported complementary and alternative medicine (CAM) use among patients with fibromyalgia and explore the associations between CAM treatments, self-reported quality of life, and pain levels [100]. The study, conducted through a web-based questionnaire, revealed that approximately 66% of respondents utilized CAM treatments, with vitamins, massage therapy, and meditation as the most common. The findings suggested that individuals combining CAM and pharmacologic treatments reported significantly higher quality of life compared to those using pharmacologic treatments alone. Additionally, respondents using only CAM treatments reported lower pain levels compared to those relying solely on pharmacologic treatments. The study concludes that a considerable proportion of fibromyalgia patients use CAM, and integrating these treatments into conventional approaches may offer beneficial effects, potentially leading to a more holistic treatment approach and improved symptom relief for these patients. The authors propose this approach as particularly relevant given the challenges patients with fibromyalgia face in accessing controlled substances. Also, chiropractic adjustments may be considered by some individuals to address musculoskeletal issues and alleviate pain [101].

## 8. Emerging Treatments and Research

Advances in neurobiological research aim to unravel the intricate mechanisms involved in fibromyalgia. Imaging studies, including functional magnetic resonance imaging and positron emission tomography (PET), provide insights into the central nervous system abnormalities associated with fibromyalgia.

Borsook et al. [102] published a study reviewing the current knowledge on brain changes in chronic pain and explores how diverse brain imaging methods have facilitated these insights. Their findings suggest that advances in brain imaging techniques have transformed our perception of chronic pain, moving from a focus on the somatosensory system to recognizing the involvement of emotional, cognitive, and modulatory areas of the brain, as well as degenerative processes and contributing to the development and persistence of pain symptoms, along with associated features like anxiety, depression, and cognitive changes.

Furthermore, genetic research continues to identify potential genetic markers associated with fibromyalgia susceptibility. This could aid in the diagnosis, assess disease severity, and guide treatment decisions [103]. Currently, there are no definitive biomarkers for fibromyalgia, but research is progressing to identify objective measures that can enhance diagnostic accuracy [103].

Glutamate, a neurotransmitter, plays a role in pain signaling. Investigational therapies targeting glutamate receptors, such as N-methyl-D-aspartate (NMDA) receptors, are being explored to modulate pain perception in fibromyalgia [104]. These include medications like ketamine, which has shown promise in early studies [104].

Cannabinoids are also being investigated for their potential analgesic and anti-inflammatory properties [105]. Some studies suggest that cannabinoids may offer pain relief and improve sleep in individuals with fibromyalgia [106].

Growing evidence links the gut microbiome to various health conditions, including fibromyalgia [107]. Investigational therapies focus on modulating the gut microbiota through dietary changes, probiotics, or fecal microbiota transplantation to potentially impact symptoms and overall well-being [108].

The integration of digital health tools, wearable devices, and telemedicine may enhance the monitoring and management of fibromyalgia [109]. These technologies can facilitate remote symptom tracking, provide real-time feedback, and improve access to healthcare resources [109].

## 9. Conclusions

In conclusion, fibromyalgia, characterized by widespread musculoskeletal pain and associated symptoms, presents a complex and evolving clinical entity. Its historical evolution from a rheumatic disorder to a recognized medical condition has been marked by skepticism within the medical community, fueled by a lack of objective diagnostic markers. Advances in research have elucidated the multifaceted nature of fibromyalgia, encompassing genetic, neurological, immunological, and psychosocial factors.

The syndrome’s impact on quality of life is profound, affecting daily activities, sleep, cognitive function, and social interactions. Diagnosis has evolved from reliance on tender points to a more comprehensive assessment, emphasizing the widespread nature of pain and associated symptoms. The risk factors, including gender predominance, genetic predisposition, and comorbid conditions, contribute to the complexity of fibromyalgia.

Management approaches involve a multidimensional strategy, including pharmacological interventions, non-pharmacological therapies, lifestyle modifications, and alternative and complementary treatments. The integration of digital health tools and emerging research on glutamate modulation, cannabinoids, gut microbiome, and genetic markers offer promising avenues for future treatments.

In navigating fibromyalgia’s intricate landscape, a holistic and patient-centered approach is crucial, recognizing the diverse manifestations and addressing the physical, emotional, and social dimensions of the condition. As research continues to unravel its mechanisms and therapeutic possibilities, a comprehensive understanding of fibromyalgia is imperative for improving patient outcomes and enhancing the overall quality of life for individuals living with this complex syndrome.

## Figures and Tables

**Table 1:** Symptom severity score calculation variables.

	No Problem	Mild	Moderate	Severe
Fatigue	0	1	2	3
Trouble thinking or remembering	0	1	2	3
Waking up tired (unrefreshed)	0	1	2	3

## Data Availability

All data are available in the presented manuscript.
